# Down‐regulation of PKM2 enhances anticancer efficiency of THP on bladder cancer

**DOI:** 10.1111/jcmm.13571

**Published:** 2018-03-07

**Authors:** Qiongli Su, Ting Tao, Lei Tang, Jun Deng, Kwame Oteng Darko, Sichun Zhou, Mei Peng, Shanping He, Qing Zeng, Alex F. Chen, Xiaoping Yang

**Affiliations:** ^1^ Key Laboratory of Study and Discovery of Small Targeted Molecules of Hunan Province Department of Pharmacy School of Medicine Hunan Normal University Changsha Hunan China; ^2^ Key Laboratory of Chemical Biology and Traditional Chinese Medicine Research (Ministry of Education of China) Hunan Normal University Changsha Hunan China; ^3^ Department of Urology Surgery 3rd Affiliated Hospital Changsha Hunan China; ^4^ School of Pharmacy Central South University Changsha Hunan China; ^5^ Department of Pharmacy Xiangya Hospital Central South University Changsha Hunan China; ^6^ Animal Nutrition and Human Health Laboratory Hunan Normal University Changsha Hunan China

**Keywords:** bladder cancer, p‐AMPK, PKM2, p‐STAT3, THP

## Abstract

Pyruvate kinase M2 (PKM2) regulates the final step of glycolysis levels that are correlated with the sensitivity of anticancer chemotherapeutic drugs. THP is one of the major drugs used in non‐muscle‐invasive bladder cancer instillation chemotherapy. However, low response ratio of THP (19.7%) treatment to human genitourinary tumours using collagen gel matrix has been observed. This study aims to investigate the effect of down‐regulation of PKM2 on THP efficiency. Via inhibitor or siRNA, the effects of reduced PKM2 on the efficiency of THP were determined in 2 human and 1 murine bladder cancer cell lines, using MTT, cologenic and fluorescence approaches. Molecular mechanisms of PKM2 on THP sensitization were explored by probing p‐AMPK and p‐STAT3 levels via WB. Syngeneic orthotopic bladder tumour model was applied to evaluate this efficiency in vivo, analysed by Kaplan‐Meier survival curves, body and bladder weights plus immunohistochemistric tumour biomarkers. PKM2 was overexpressed in bladder cancer cells and tissues, and down‐regulation of PKM2 enhanced the sensitivity of THP in vitro. Activation of AMPK is essential for THP to exert anti‐bladder cancer activities. On the other hand, down‐regulating PKM2 activates AMPK and inhibits STAT3, correlated with THP sensitivity. Compared with THP alone (400 μmol L^−1^, 50 μL), the combination with metformin (60 mmol L^−1^, 50 μL) stopped growth of bladder cancer completely in vivo (combination group VS normal group *P* = .078). Down‐regulating the expression of PKM2 enhances the anticancer efficiency of THP. This study provides a new insight for improving the chemotherapeutic effect of THP.

## 1 INTRODUCTION

Cancer is a major public health issue worldwide. Bladder cancer falls in the seventh place in male incidence of cancer and increases yearly.^1^ Even though transurethral resection has been served as the standard treatment, recurrence and metastasis are often seen in clinic.^2^ The commonest way to prevent recurrence and progression is to supplement transurethral resection with intravesical chemotherapy or immunosuppressive agents.^3^, ^4^ No substantial progress over the past 3 decades has been reported in clinical administration for bladder cancer except FDA‐approved PD‐L1 treatment in 2016.^5^

Pirarubicin (THP, C32H37NO12) has a favourable antitumour efficiency with limited side effects; the potential application of THP in solid tumours has aroused great interest among researchers. Currently, it is one of the major drugs used in non‐muscle‐invasive bladder cancer instillation chemotherapy to improve chemotherapeutic efficacy. However, low response ratio of THP (19.7%) treatment to human genitourinary tumours using collagen gel matrix has been observed.^6^ Furthermore, molecular mechanisms underlying this low response ratio are still not clear. Several scientists have demonstrated that when STAT3‐specific siRNA knock‐downs STAT3 expression or inhibitor (ie Stattic) inhibits STAT3 activity, it decreases the IC50 of THP treatment dramatically in bladder cancer cells.^7^ This indicates that STAT3 plays an important role in the pharmacodynamics of THP. STAT3 is a member of the STAT family of transcription factors and is activated in several cancers including osteosarcoma,^8^ renal cell carcinoma,^9^ and pancreatic cancer.^10^ Inhibition of STAT3 could serve as a potent chemotherapeutic sensitizing strategy to overcome drug resistance in chemoresistant cancer cells.^11^, ^12^ However, STAT3 is not an ideal molecular target for cancer therapy, because of its potential damage to normal tissue and other off‐target effects. Gao et al^13^ showed that PKM2 regulates constitutive activation of STAT3 in CRC cells. In this study, we are the first to observe that the expression of PKM2 in bladder cancer cell lines UMUC3, T24 and MB49 is inversely correlated with the IC50 values of THP treatment. This novel finding indicates that regulating PKM2 expression may be a valid strategy to enhance the efficiency of THP administration. Pyruvate kinase (PK) regulates the final rate‐limiting step of glycolysis and catalyses the transfer of a phosphate group from phosphoenolpyruvate (PEP) to adenosine diphosphate.^14^ This transfer yields one molecule each of pyruvate and adenosine triphosphate.^15^, ^16^ PKM1, PKM2, PKL and PKR are PK isoforms expressed in different types of mammalian cells and tissues. Alternate splicing of PKM pre‐mRNA by hnRNP A1/2 and polypyrimidine‐tract binding (PTB) protein splicing factors leads to PKM2 generation by including exon 10 and excluding exon 9.^17^, ^18^, ^19^ PKM2 has been an attractive target for cancer therapy because of its high levels of expression in most carcinomas.^20^ Recent research strongly suggests that PKM2 plays an important role in the production of low‐grade non‐invasive and high‐grade invasive urothelial carcinomas.^21^ According to recent findings, high expression of PKM2 was associated with chemosensitivity to epirubicin and 5‐fluorouracil in breast cancer and also enhanced chemosensitivity to cisplatin in cervical cancer.^22^, ^23^ PKM2 has also shown contribution to gefitinib resistance via up‐regulation of STAT3 activation in colorectal cancer.^24^ Depletion of PKM2 led to apoptosis or sensitivity of several tumour cells to chemotherapy.^25^, ^26^ However, whether PKM2‐induced STAT3 phosphorylation plays a significant role in the regulation of THP sensitivity in bladder cancer cells remains unclear.

PKM2 is closely related to metabolism. Our previous study has demonstrated that activated AMPK increases the efficacy of gefitinib on bladder cancer cells, related to metabolism‐associated Warburg effect.^27^ Furthermore, Li et al^28^ showed that PKM2 inhibitor reverses AMPK activity. Thus, in this study, we aim to explore anticancer effect of THP and its molecular mechanisms on bladder cancers after down‐regulating PKM2, focusing on the roles of p‐STAT3 and p‐AMPK.

## 2 MATERIALS AND METHODS

### 2.1 Reagents

THP and Compound C were purchased from Selleck (Houston, TX, USA). Metformin was purchased from Aladdin chemistry Co. Ltd (Houston, TX, USA). They were diluted across a range of concentrations in culture media. Antibodies against PKM2, phosphor‐STAT3(Y705), total STAT3, phosphor‐AMPKα, total AMPKα and β‐actin were purchased from Cell Signaling Technology (Cell Signaling, Beverly, MA, USA).

### 2.2 Cell lines and culture conditions

Murine and human bladder cancer cell lines provided by Dr. P Guo were cultured in DMEM supplemented (Hyclone, Logan, UT, USA) with 10% of FBS (Hyclone, Logan, UT, USA) and 1% of penicillin‐streptomycin at 37°C, in humidified air containing 5% of CO_2_.

### 2.3 Human urothelial carcinoma tissues

Adjacent normal tissues (n = 6) and urothelial carcinoma tissues (n = 10) from patients who underwent transurethral resection or radical cystectomy of urothelial carcinoma were collected, following a protocol approved by both Institutional Review Boards of Xiangya Third Hospital, Central South University and Hunan Normal University. De‐identified fresh tissues were fixation, paraffin‐embedding and sectioning for immunohistochemistry. For scoring of immunohistochemical staining of human specimens, both the proportion and the intensity of the positive staining were scored following published methods,^29^ with the proportion graded in 6 scales (0‐5; ie, 0, none; 1,<1/100; 2, 1/100 to 1/10; 3, 1/10 to 1/3; 4, 1/3 to 2/3; and 5, more than 2/3), and the intensity graded in 4 scales (0‐3; 0, none; 1, weak; 2, intermediate; and 3, strong). The total score from 0 to 8 was computed by combining the proportion and the intensity scores.

### 2.4 Cell viability and cologenic assay

Cell viability was assessed using a tetrazolium‐based assay using microplate reader (Biotek, SYNERGY HTX, Vermont, USA). IC_50_ values were determined through the dose‐response curves.

Cologenic survival was defined as the ability of the cells to form colonies. Images were taken and analysed by microscopy (Leica, DFC450C; Wetzlar, Germany) and microplate reader (Biotek, SYNERGY HTX).

### 2.5 Assessment of apoptosis

Apoptosis was analysed by fluorescence microscopy (Leica, DFC450C). First, a total of 1.2 × 10^4^ cells were seeded onto 96‐well culture plates. After 24 hours, parental and PKM2 siRNA cells were treated with THP alone or combination, respectively. Another 24 hours later, 100 μL binding buffer, 1 μL of Annexin V‐FITC and 1 μL of propidium iodide were added into cells at room temperature in the dark for 15 minutes, kept in a 4°C temperature, and then examined and collected images under fluorescence microscopy. Images were taken by microscopy (Leica, DFC450C) and semi‐quantitative analysis with Image J; the average optical density indicates the fluorescence intensity; and the higher the average optical density, the more the number of apoptotic cells.

### 2.6 Protein characterization

Western blot assessment was performed with regular procedure. Primary antibody was added in BSA and allowed to incubate overnight at 4°C, washed with TBS/0.1% Tween‐20 for 5 times (10 minutes per time) before the secondary antibody was added and then incubated for an additional hour at room temperature. The membrane was again washed 3 times before adding Pierce Super Signal chemiluminescent substrate (Rockford, IL, USA), then immediately imaged on Chemi Doc (Bio‐Rad, Hercules, CA, USA) and quantified by Image J (NIH, Bethesda, MD, USA).

### 2.7 STAT3 and PKM2 knock down by transient transfection

Gene silencing of PKM2 and STAT3 in UMUC3 was completed by siRNA techniques. Inhibition of PKM2 mRNA translation was performed with siRNA targeted to PKM2 mRNA (Sense strand: 5′ CCAUAAUCGUCCUCACCAAUU 3′; THERMO scientific), with the transfection reagent Lipofectamine 2000 (Invitrogen). Sequence of siRNA targeted to STAT3 mRNA: Sense strand: 5′ CAUCUGCCUAGAUCGGCUAdTd 3′. Control siRNA (Ribobio) was transfected into UMUC3 alongside siRNA experiments as a negative control. Briefly, 3 × 10^5^ UMUC3 were seeded in six‐well plates. UMUC3 cells at 30%‐50% confluence were transfected with Lipofectamine 2000 and alongside siRNA (at a final concentration of 50 nmol) or control siRNA in the absence of antibiotics or FBS for 6 hours. Following a washing in phosphate‐buffered saline (PBS), the medium was replaced with DMEM for an additional 72 hours. Cell protein and total RNA were collected for analysis. Specific silencing was confirmed by Western blot.

### 2.8 Cross‐linking

For the cross‐linking of PKM2 in cells, UMUC3 cells were seeded into 6‐well plates and cultured in normal conditions [DMEM supplemented with 10% (vol/vol) FBS], MEM supplemented with metformin in different concentration (0, 2 mmol L^−1^) for 36 hours each. The cells were washed 3 times with PBS to remove serum proteins and then incubated on culture plates with a hypotonic buffer (PBS diluted by a factor of 10) for 15 minutes. After 3 washes with the hypotonic buffer, the cells were cross‐linked with the homobifunctional N‐hydroxysulfosuccinimide‐ester cross‐linker, DTSSP (Thermo Fisher Scientific) at room temperature for 30 minutes (pH7‐9), according to the manufacturer's protocol. The treated cells were lysed with an equivalent amount of concentrated RIPA buffer [50 mmol L^−1^ Tris·HCl (pH 7.8 or pH8.6), 300 mmol L^−1^ NaCl,1% Nonidet P‐40] and successively quenched with 10 mmol L^−1^ Tris·HCl for an additional 15 minutes. After centrifugation at 14 000×*g* for 15 minutes, the supernatant was collected.

### 2.9 Animals

Female C57BL/6 mice were purchased from Hunan SJA Laboratory Animal Co., Ltd (Changsha, Hunan, China). Animals were housed 4 per cage in a specific pathogen‐free animal facility. The experimental protocol was reviewed and approved by the Institutional Animal Care and Use Committee at Hunan Normal University.

### 2.10 Orthotopic implantation and intravesical treatment

Exponential growth of MB49 cells^30^ was harvested, and cell density in collection tube was counted by cell counter. Female mice 6 to 8 weeks of age were used for cancer cell implantation. Briefly, 1.2 × 10^5^ MB‐49 cells in 0.1 mL PBS were injected into the bladder wall using 1‐mL syringes and catheter scratching according to the previously described protocol.^31^

A total of 5 groups designed are Normal Female C57BL/6 mice which received intravesically an instillation of 50 μL of PBS as control group, Female C57BL/6 mice with orthotopic bladder cancer were randomly divided into 4 groups, which received intravesically an instillation of 50 μL of PBS metformin (60 mmol L^−1^, 50 μL, twice per week), THP (400 μmol L^−1^, 50 μL, twice per week), or combinations of metformin with THP‐treated group (metformin 60 mmol L^−1^, THP 400 μmol L^−1^, total 50 μL, twice per week). Each group has 12 Female C57BL/6 mice. All treatments started at day 2 post‐tumour implantation for 2 weeks.

### 2.11 Histological analysis

Tissue processing, PKM2, p‐STAT3, haematoxylin and eosin and Ki67 staining of 7‐μm tissue sections were conducted by Department of Pathology, Hunan Provincial Cancer Hospital, Changsha, Hunan, P. R. China. The slides were reviewed by a pathologist, Dr. Lei Xue. The pathology evaluation was performed to confirm the presence or absence of tumour. Analysis of images was carried out using Image Pro Plus 4.5 software. The mean density was used as a semi‐quantitative measure of relative immunoreactivity.

### 2.12 Statistical analysis

All data are presented as mean ± SD. Statistical analyses were carried out using ANOVA, and statistical significance was assumed at a value of *P* < .05.

## 3 RESULTS

### 3.1 PKM2 is highly expressed in bladder tumour tissue and tumour cells

**Figure 1:**
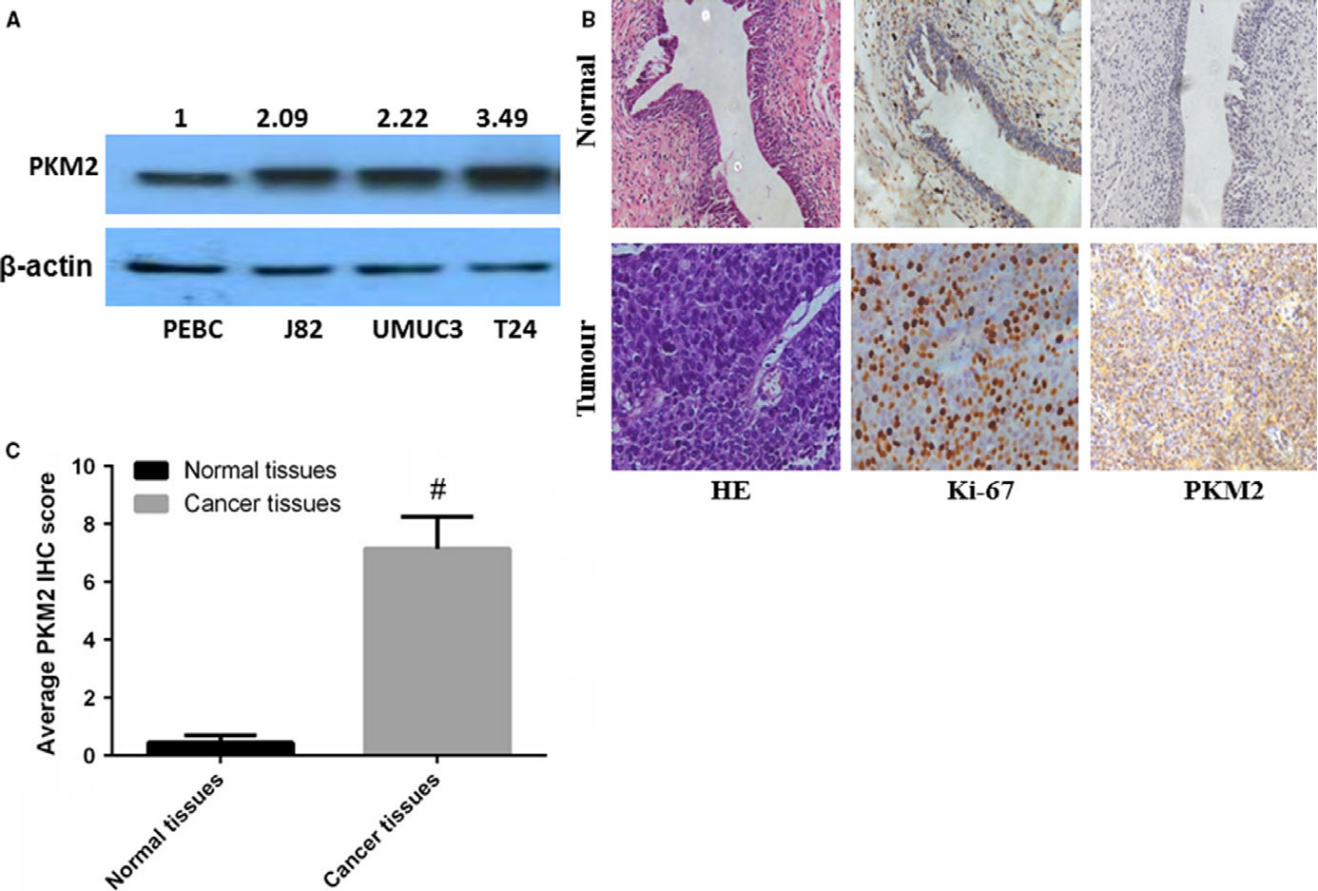
PKM2 is highly expressed in bladder tumour tissue and tumour cells. A, PKM2 expression was quantitated in 3 human bladder cancer cells and one normal bladder cell using WB; B, the expression of PKM2 in tissues was evaluated using immunohistochemistry. Two groups of specimens (N = 6 and 10 for adjacent normal tissues and cancer tissues, respectively) were paraffin‐embedded, sectioned and immunohistochemically stained with anti‐PKM2. Haematoxylin and eosin and Ki‐67 were used to indicate whether there is a tumour. C, The differences between normal and tumour tissues regarding the expression levels of PKM2 were statistically significant (*P < .05, ^#^ *P* < .01)

We surveyed several established cell lines and found that PKM2 was highly expressed in human bladder cancer cell lines than primary‐cultured human normal urothelial cell PEBC (Figure 1A). As shown in Figure 1B,C, PKM2 was undetectable in normal urothelia tissues but highly expressed in urothelial carcinoma tissues by immunohistochemistry. These results are consistent with another observation that PKM2 is closely related to the development of bladder cancer.^21^

### 3.2 THP inhibits bladder cancer cell proliferation via activating AMPK, and this inhibitory effect is closely related to PKM2

**Figure 2:**
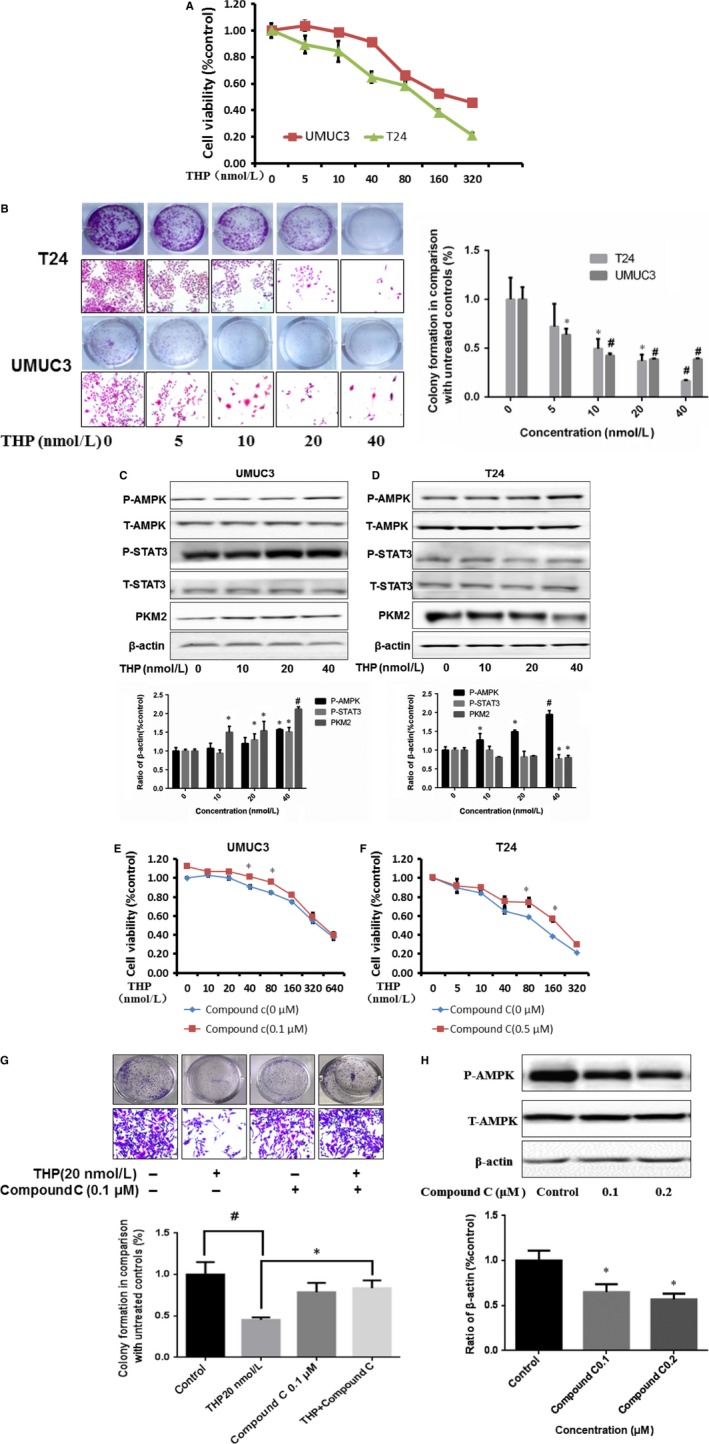
THP inhibits bladder cancer cell proliferation via activating AMPK and closely related with PKM2. A, T24 and UMUC3 were treated with THP (0, 5, 10, 40, 80, 160 and 320 nmol L^−1^), cells viability were measured by MTT assays after 72 hours incubation; B, T24 and UMUC3 were treated with THP (0, 5, 10, 20 and 40 nmol L^−1^), cell numbers were measured by colony formation assay after 7 days incubation and took photographs by microscopy; C, Western blotting analysis showing that the expression levels of PKM2/p‐STAT3 and p‐AMPK were significantly up‐regulated after THP treated in UMUC3. Results are presented as the median of 5 independent experiments; D, Western blotting analysis showing that the expression levels of PKM2/p‐STAT3 were significantly up‐regulated but activities of p‐AMPK were decreased after THP treated in T24. Results are presented as the median of 5 independent experiments; E‐F, MTT (cell viability) was assessed with 72 hour THP alone or THP combined with 0.1 μmol L^−1^ AMPK antagonist Compound C treatment in UMUC3 (E) and 0.5 μmol L^−1^ AMPK antagonist Compound C treatment in T24 (F); G, UMUC3 were treated with THP(20 nmol L^−1^) and Compound C(0.1 μmol L^−1^) alone or combination, cell numbers were measured by colony formation assay after 7 days incubation and took photographs by microscopy; H, Western blotting analysis showing that the expression levels of p‐AMPK after treating by Compound C on UMUC3 cell line. Results are presented as the median of 5 independent experiments; statistical analyses were carried out using *T*‐test analysis (*P < .05, ^#^ *P* < .01)

Two human bladder cell lines were exposed to 0‐640 nmol L^−1^ THP. THP exhibited a dose‐dependent inhibition of cell proliferation. The IC50 values were 146.80 and 247.24 nmol L^−1^ in T24 and UMUC3, respectively **(**Figure 2A, Table 1). We next examined colony formation and found that THP exhibits a dose‐dependent inhibitory effect at concentration range from 0 to 40 nmol L^−1^ (Figure 2B). THP had a stronger inhibitory effect on T24 than on UMUC3 with similar pattern observed in proliferation assay described above. In order to clarify why different bladder cancer cells had different sensitivities to THP, we examined the PKM2 protein levels among the two cell lines after treatment with THP. The results revealed that THP reduced PKM2 expression in T24 and increased that of UMUC3. This novel finding implies that PKM2 is a key regulator to THP efficacy. Simultaneously, we discovered that AMPK was activated after THP treatment in UMUC3 (Figure 2C) and T24 (Figure 2D). In order to further determine whether the inhibition of p‐AMPK is associated with the reduced efficacy of THP, we combined Compound C, an inhibitor of p‐AMPK with THP, and found a significant antagonistic effect in both UMUC3 (Figure 2E) and T24 (Figure 2F) via MTT assay. In addition, UMUC3 cell lines treated by THP and Compound C also showed antagonism in cloning experiments (Figure 2G). After treatment by Compound C in different concentrations, the expression of p‐AMPK showed a concentration‐dependent decrease in UMUC3 cell line (Figure 2H), indicating the role of activated AMPK in efficacy of THP. Thus, we demonstrated that this inhibitory effect of THP is closely related with PKM2 via activating AMPK.

**Table 1:** Inhibitory concentration 50% (IC_50_) for THP

Cells	T24	UMUC3
IC_50_ (nmol L^−1^)	146.80	247.24

### 3.3 Down‐regulation of PKM2 enhanced the inhibitory effect of THP via inhibiting p‐STAT3

**Figure 3:**
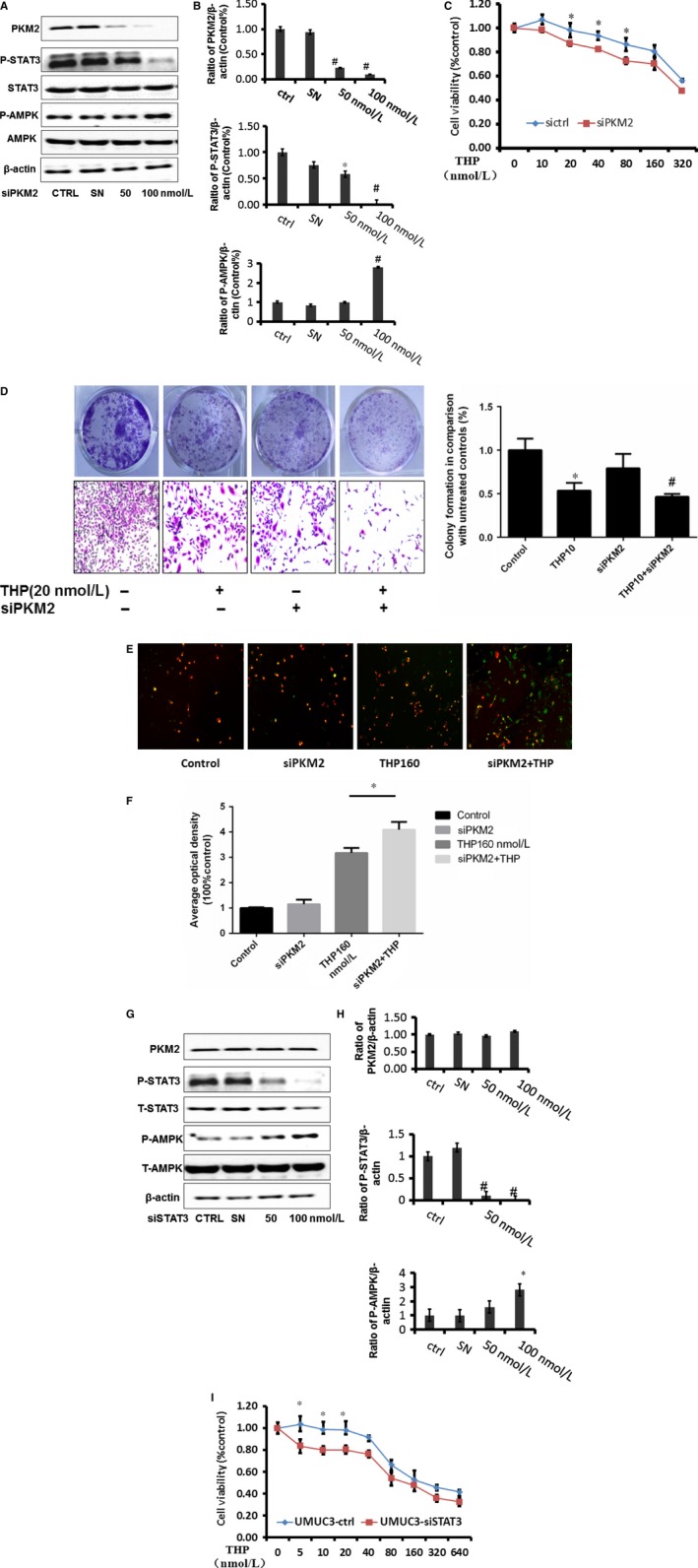
Down‐regulation of PKM2 enhanced the inhibitory effect of THP via inhibit p‐STAT3 and activated p‐AMPK. A‐B, The expressions of PKM2, p‐STAT3 and p‐AMPK after knocking down PKM2 in UMUC3, SN represents control siRNA (Ribobio). Represent the ratio of proteins of interests to β‐actin was calculated by the band density of Western blots of UMUC3 cell line using Image J software. (*P < .05, ^#^ *P* < .01); C, MTT was assessed with 72 hour THP alone or THP combined with siPKM2 treatment on UMUC3 cell proliferation; D, colony formation assay results of siPKM2 combined with THP(20 nmol L^−1^) treatment on UMUC3 with 7 days treatment; E‐F, immunofluorescence results of siPKM2 combined with THP(160 nmol L^−1^) treatment on UMUC3 cell apoptosis; G‐H, protein expressions of p‐STAT3, PKM2 and p‐AMPK after knocking down STAT3 in UMUC3, SN represents control siRNA (Ribobio); represent the ratio of proteins of interests to β‐actin was calculated by the band density of Western blots of UMUC3 cell line using Image J software (*P < .05, ^#^ *P* < .01). (I) MTT assay results of siSTAT3 combined with THP (20 nmol L^−1^) treatment on UMUC3. Results are presented as the median of 5 independent experiments

These novel findings described above inspired us to further probe the relationship between THP efficacy and the expression of PKM2. We knocked down PKM2 (Figure 3A‐B) and found that down‐regulation of PKM2 enhanced the inhibitor effect of THP on bladder cancer cells detected by MTT (Figure 3C), colony formation assay (Figure 3D) and fluorescence microscopy approach (Figure 3E), indicating the inverse role of PKM2 on THP sensitivity to bladder cancer cells. Furthermore, knocking down of STAT3 enhanced the effect of THP on bladder cancer cells as well detected by MTT (Figure 3H). Meanwhile, p‐STAT3 was significantly reduced when PKM2 was knocked down. However, knocking down STAT3 did not change the expression of PKM2 (Figure 3F‐G). The metabolic marker AMPK was activated after knocking down both PKM2 and STAT3. These results indicated that down‐regulation of PKM2 inhibits STAT3 activation. Meanwhile, knocking down of either PKM2 or STAT3 significantly activates AMPK. Thus, down‐regulation of PKM2 is probably through the inhibition of p‐STAT3 and activation of AMPK to achieve the purpose of enhancing efficacy.

### 3.4 Metabolism modulator—metformin inhibited PKM2

**Figure 4:**
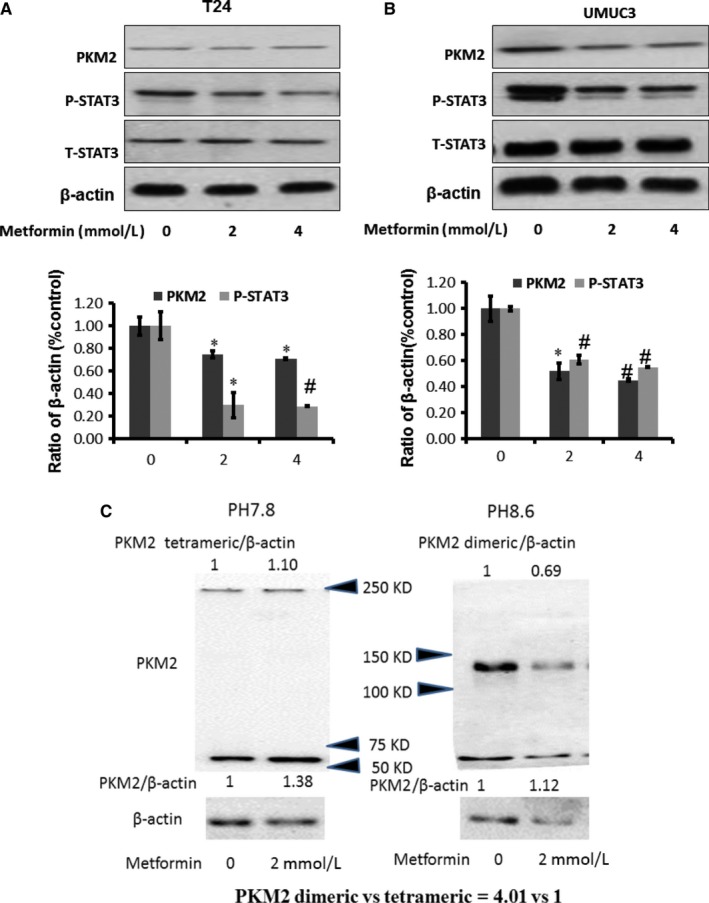
Metabolism modulators—metformin inhibits PKM2. A‐B, Metformin inhibits PKM2 and p‐STAT3 in T24 and UMUC3, respectively. β‐actin was included as a loading control. The ratio of proteins of interests to β‐actin was calculated by the band density of Western blots of T24 and UMUC3 cell lines using Image J software (*P < .05, ^#^ *P* < .01). Results are presented as the median of 5 independent experiments. C, Western blot analysis of PKM2 using anti‐PKM2 antibody. UMUC3 cells were cultured in medium containing metformin (0, 2 mmol L^−1^) and treated with a cross‐linker. The results of the Western blot indicate that dimer formation is much more than PKM2 tetramer in the UMUC3 cells (4.01:1). And dimer formation in the cells was decreased after exposure to metformin

Metformin, a widely prescribed drug for treating type II diabetes, is one of the most extensively recognized metabolic modulators, with activating AMPK.^32^ Metformin showed inhibitory effect in various cancer cell lines and xenograft cancer models and sensitized chemotherapy drugs.^33^ Our recent study has demonstrated that metformin alone inhibited bladder tumour growth and increased the efficacy of gefitinib via activating AMPK.^34^ To determine the role of metabolic modulators, we observed that metformin inhibited PKM2 expression obviously (Figure 4A‐B). PKM2 has monomer, dimer and tetramer forms under cross‐linking conditions.^34^ Dimer is active form of PKM2, and tetramer is inactive one.^35^ Using cross‐linker DTSSP, we are able to detect dimer and tetramer at pH 8.6 and pH 7.8, respectively (Figure 4C). At basal level (control), dimer formation is more than PKM2 tetramer (Figure 4C). The formation of dimers in the cells was decreased while slight increase in tetramer after exposure to metformin, indicating the decreased activities of PKM2, consistence with the results in the absence of cross‐linkers (Figure 4C).

### 3.5 THP and metformin synergistically inhibited bladder cancer growth in vitro

**Figure 5:**
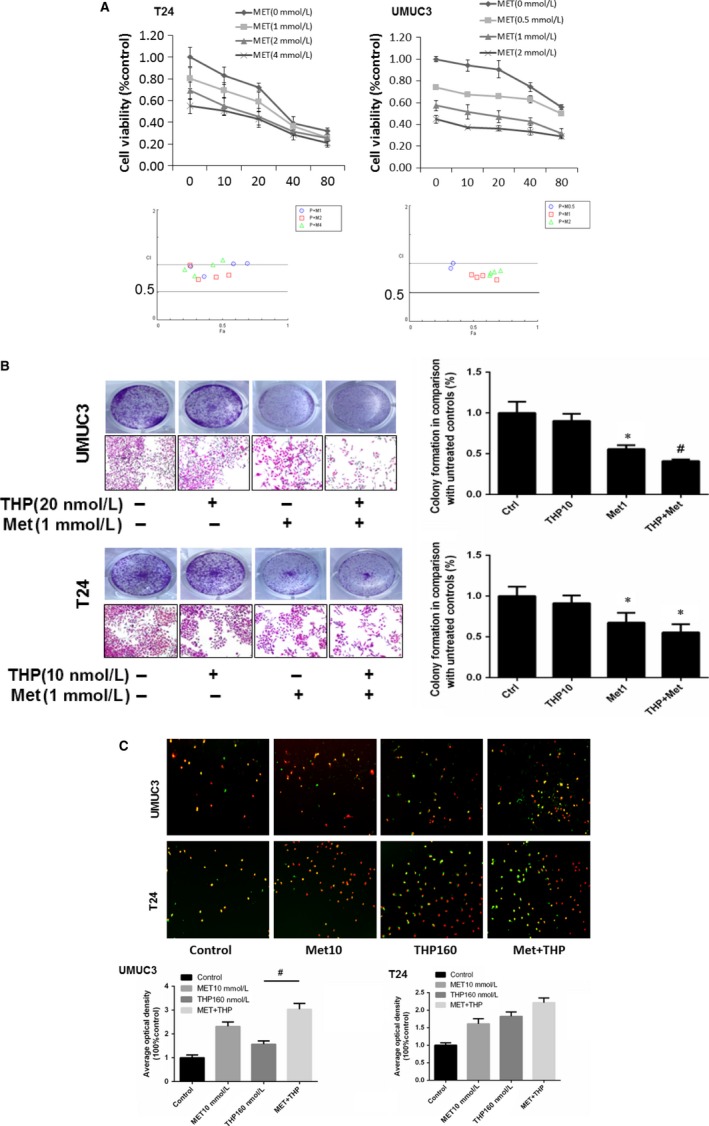
THP and metformin synergistically inhibited bladder cancer growth in vitro. A, THP and metformin synergistically inhibited bladder cancer growth determined by MTT. Below: combination index (CI) among the combinations of 2 drugs was calculated using CompuSyn software. If CI = 1, it denotes additives; if CI >1, it denotes antagonism; if CI <1, it denotes synergism. CI values in the vast majority of combinations were less than 1, indicating synergism. Results are presented as the median of 5 independent experiments. B, T24 and UMUC3 were treated by Met(1 mmol L^−1^) and THP (20 nmol L^−1^ in UMUC3 and 10 mmol L^−1^ in T24) alone or combination; cell numbers were measured by colony formation assay after 7 days incubation. C, Met (10 mmol L^−1^) and THP (160 nmol L^−1^) alone or combination treated T24 and UMUC3 to indicate cell apoptosis via immunofluorescence experiments**.** Results are presented as the median of 5 independent experiments (*P < .05, ^#^ *P* < .01)

Generally, at the range of tested concentrations (0‐80 nM), compared with THP treatment alone, combination of THP with metformin exhibited a synergistic effect as assessed by CompuSyn software (Fig 5A). Colony formation assay showed that the inhibitory effect of THP increased when combined with metformin (Figure 5B). Cellular apoptosis was considerably increased when combined with metformin at 24 hours detected by fluorescence microscopy (Figure 5C).

Taken together, these results have demonstrated that the combined use of THP and metformin synergistically inhibited proliferation and colony formation of bladder cancer cells.

### 3.6 Treatment of THP alone or combined with metformin on murine bladder cancer cell MB49

**Figure 6:**
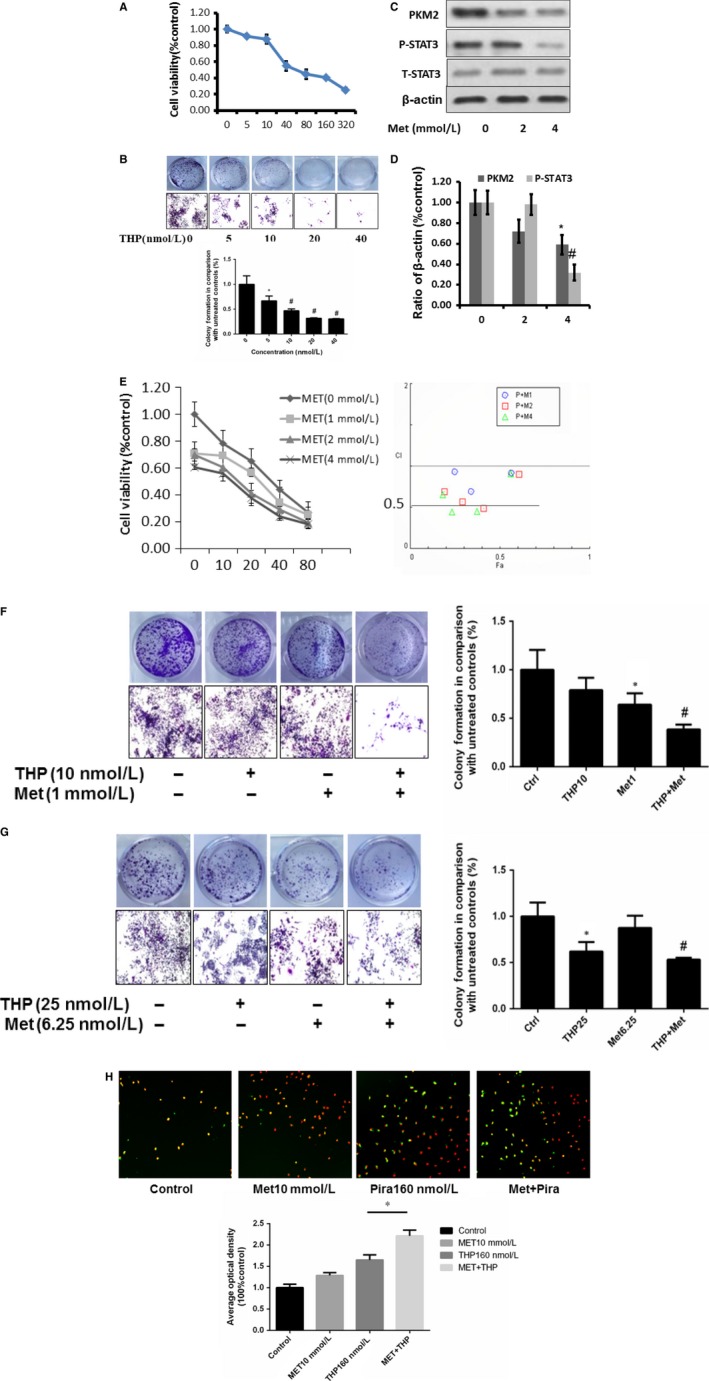
Simulation of animal experiments. A, MTT results of THP treatment on cell proliferation of murine bladder cancer cell line MB49; B, colony formation assay results on MB49 with 7 days treatment at different concentrations (0, 5, 10, 20, 40 nmol L^−1^); C‐D, effects of THP on PKM2/p‐STAT3 inMB49 after Met(0, 2, 4 mmol L^−1^) treatments. Represent the ratio of proteins of interests to β‐actin was calculated by the band density of Western blots of MB49 cell line using Image J software (*P < .05, ^#^ *P* < .01); E, THP and metformin synergistically inhibited bladder cancer growth determined by MTT. Below: combination index (CI) among the combinations of 2 drugs was calculated using CompuSyn software. If CI = 1, it denotes additives; if CI >1, it denotes antagonism; if CI <1, it denotes synergism. CI values in the vast majority of combinations were less than 1, indicating synergism; F, MB49 was treated by Met(1 mmol L^−1^) and THP (10 nmol L^−1^) alone or combination; cell numbers were measured by colony formation assay after 7 days incubation; G, synergism effect of THP and metformin on MB49 via colony formation assay at labelled concentrations, twice per week for 2 weeks, was observed. H, Met(10 mmol L^−1^) and THP (160 nmol L^−1^) alone or combination treated MB49 to indicate cell apoptosis via immunofluorescence experiments. Results are presented as the median of 5 independent experiments

Since MB49 is the most popularly used murine bladder cancer cell line to establish syngeneic orthotopic bladder cancer model, we examined the inhibitory effect of THP alone or combined with metformin in MB49. As shown in Figure 6A, the IC50 value of THP in MB49 is 140.07 nmol L^−1^. We also examined colony formation at concentration range from 0 to 40 nmol L^−1^. It was found that THP exhibits a dose‐dependent inhibitory effect in MB49 (Figure 6B). We found that metformin inhibited PKM2 expression (Figure 6C‐D) similar to results showed by T24 and UMUC3. MTT and cologenic assays indicated that the combination of metformin with THP enhanced the inhibitory effect of THP. Similar results were observed in UMUC3 and T24 (Figure 6E‐G). Furthermore, an intermittent treatment to MB49 with THP alone or combined with metformin for 2 hours was used to mimic intravesical treatment. Cells were treated twice per week for 2 weeks in vitro. The results demonstrated that combination of THP with metformin significantly reduced colony formation in MB49 at the indicated concentrations (Figure 6H).

### 3.7 Intravesical treatment of THP and metformin exerted potent anticancer effects on orthotopic bladder tumours

Orthotopic mouse model was established to provide the useful tool to determine the effect of intravesical localized treatment.^31^ Syngeneic tumour implantation provided better tumour take rate compared to the xenograft implantation.^36^ We reached 100% tumour take rate using previously reported technique.^31^

**Figure 7:**
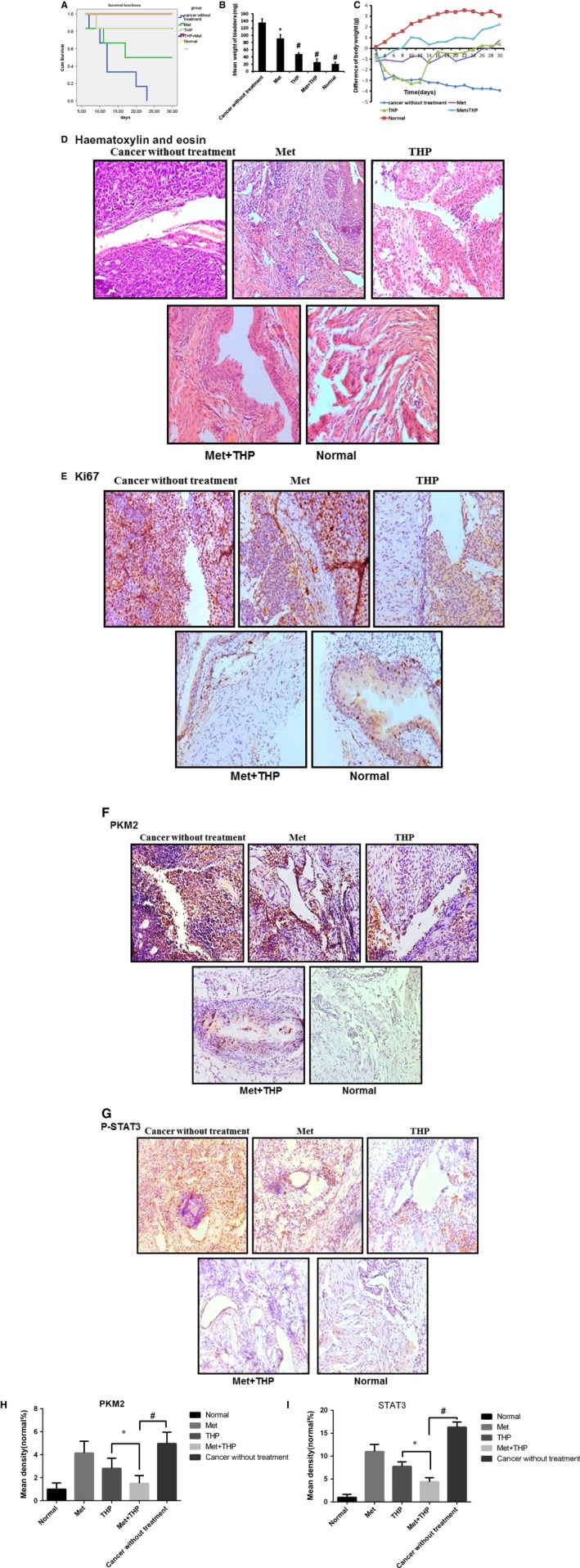
THP and metformin synergistically inhibited bladder cancer growth in vitro intravesically. A, Kaplan‐Meier survival analysis of 5 groups. Death of mice was checked daily, and cumulative survival rate was plotted against the time course. The average lifespan in group I was only 12.5 days. Respectively, all living mice were killed at day 30. B, Weights of mouse bladders including those died before the end of experiment were measured. C, The weight of mice was measured daily. All bladder tissues were collected and fixed. D‐E, Histological sections from these tissues were subjected to haematoxylin and eosin stain or immunohistochemistry for Ki67 to confirm the presence or absence of tumours. F‐G, Immunohistochemistry was used to detect the expression of PKM2 and p‐STAT3 of bladder tissues from mice of 5 groups. H‐I, Mean density of PKM2 and STAT3 was selected and counted on images using Image Pro Plus 4.5 software (*P < .05, ^#^ *P* < .01)

The antitumour effect of THP was evaluated after combination with metformin using an orthotopic bladder cancer model in mice. Figure 7A shows cumulative survival curves of 5 groups. Cancer cell implantation induces death of mice (group I) but intravesical treatment of metformin and THP dramatically enhanced lifespan with better survival (combination group vs tumour without treatment group, *P* = .005). Treatment with either metformin or THP alone improved mice survival, respectively. The size of the tumour at the end of the experiments was measured and used to evaluate the antitumour effect. As shown in Figure 7B, the bladder weights in normal mice group were bigger than those in other groups. Intravesical treatment with THP or metformin alone profoundly diminished bladder weights. The bladder weights between combination of metformin and THP group with normal group have no significant differences (combination treatment group vs normal mice group *P* = .078). Figure 7C demonstrated the decrease of body weight in tumour without treatment, indicating the toxicity induced by tumour implantation as we expected. This decrease in body weight is attenuated by intravesical treatment with metformin or THP, and the combination of metformin with THP is able to reverse this decrease. At the beginning of intravesical instillation in THP treatment group, the decrease in body weight was much more than the tumour group, indicating the obvious toxic side effects of THP. In contrast, we did not observe similar phenomenon in the combination treatment group. This result suggests a profound function that metformin can effectively reduce the toxic side effects caused by THP. Furthermore, haematoxylin and eosin results show the complete absence of tumour in combination treatment group (Figure 7D), and the results of Ki67 staining demonstrate similar efficiency pattern (Figure 7E). Altogether, intravesical treatment of combined drugs exhibits potent anticancer effect.

## 4 DISCUSSION

Bladder cancer remains one of the main malignancies that affect the genitourinary tract.^37^, ^38^ The main clinical treatment is surgical resection assisted by chemotherapy. In fact, the unsolved severe problem of treating this disease is chemoresistance after surgical resection. THP is one of the major clinically used drugs to treat bladder cancer via instillation administration. However, low response rate and side effect largely limit its efficacy. Thus, better personalized selection of patients for treatment could greatly improve the clinical application of this drug. Herein, we demonstrate that PKM2 overexpression in bladder cancer is one of the critical mechanisms for intrinsic resistance to THP. This study brings to light that down‐regulating the expression of PKM2 enhances the efficacy of THP on bladder cancer cell apoptosis and proliferation. Mechanically, THP induces bladder cancer cells apoptosis via DNA damage.^39^ On the other hand, down‐regulating the expression of PKM2 suppresses cancer cells proliferation and leads to DNA damage.^40^ This may be an efficient option to overcome the resistance and side effects of THP.

AMPK is activated allosterically by an increase in the intracellular AMP/ATP ratio. The most studied aspect of AMPK function is its role in maintaining cellular energy stores and regulating whole‐body energy balance.^41^ In the present study, we are the first to demonstrate that THP activated AMPK and the activation of AMPK is essential for THP to exert anti‐bladder cancer. At the same time, we also find that AMPK is significantly activated after knocking down either PKM2 or STAT3. We confirm that activating AMPK signalling pathway is involved in down‐regulating PKM2 to exert antitumour effect.

We also reveal that metformin, an AMPK activator, reduces the expression of PKM2, and the combination of THP and metformin induces stronger inhibition on proliferation, colony formation and apoptosis. In vivo studies demonstrate the consistent efficiency obtained in Petri dish study suggesting that the combination of THP with metformin may be a potential strategy for the treatment of bladder cancer.

In summary, we demonstrated the decreased PKM2 enhanced the efficiency of THP on bladder cancer via inhibiting STAT3 and activating AMPK. PKM2 could represent a novel protein marker for intrinsic THP resistance in bladder cancer patients. Targeting PKM2 may be an ideal approach for the treatment of THP‐resistant settings. Moreover, as STAT3 mediates PKM2's control of THP resistance, small molecules that disrupt the interaction of PKM2 with STAT3 might enhance THP‐induced growth inhibition and apoptosis and enhance the efficacy of THP therapy.

## CONFLICT OF INTEREST

The authors declare no commercial or financial conflict of interest.

## AUTHORS' CONTRIBUTIONS

Conception and design: Q. Su, M. Peng, A. F Chen, X. Yang; Development of methodology: Q. Su, T. Tao, S. He, J. Deng, L. Tang; Acquisition of data (provided animals, acquired and managed patients, provided facilities, etc.): Q. Su, T. Tao, J. Deng, S. Zhou, L. Tang, M. Peng, S. He; Analysis and interpretation of data (eg statistical analysis, biostatistics, computational analysis): Q. Su, T. Tao, M. Peng, S. He, Q. Zeng, A. F Chen, X. Yang; Writing, review and/or revision of the manuscript: Q. Su, A. F Chen, X. Yang, Kwame Oteng Darko; Administrative, technical or material support (ie reporting or organizing data, constructing databases): Q. Su, T. Tao, J. Deng, L. Tang, M. Peng, X. Yang.
